# Microsatellite Instability assessment in Black South African Colorectal Cancer patients reveal an increased incidence of suspected Lynch syndrome

**DOI:** 10.1038/s41598-019-51316-4

**Published:** 2019-10-21

**Authors:** M. McCabe, Y. Perner, R. Magobo, P. Magangane, S. Mirza, C. Penny

**Affiliations:** 1 Department of Anatomical Pathology, School of Pathology, Faculty of Health Sciences, University of the Witwatersrand, Parktown, Johannesburg, 2193 South Africa; 2 Department of Internal Medicine, Faculty of Health Sciences, University of the Witwatersrand, Parktown, Johannesburg, 2193 South Africa

**Keywords:** Colon cancer, DNA mismatch repair, Epidemiology

## Abstract

Microsatellite Instability (MSI) is a hallmark of colorectal cancer (CRC) and occurs in 15–16% of CRC. Molecular biological information of CRC in South Africa (SA) is largely unrecorded. This study was undertaken to determine the frequency of MSI, with particular reference to Lynch syndrome (LS) with a view to improve surveillance and prevention strategies. This was a retrospective study on CRC samples diagnosed between 2011–2015 at Charlotte Maxeke Johannesburg Academic Hospital (CMJAH). Samples diagnosed between 2011–2012 were screened for MSI by PCR and mismatch repair (MMR) immunohistochemistry (IHC), and additional BRAFV600E mutational analysis performed. T-tests, Fischer’s exact and Chi square statistical tests were applied. Twelve percent of patients displayed MSI, with increased frequency in black (15%) versus other ethnic group (OEG) (8%) patients. MSI patients were significantly younger than microsatellite stable (MSS) patients, however when stratified by ethnicity, black patients were predominantly younger (median age: 47), with increased MSH2/6 loss, and no BRAF mutations. These findings suggest a large proportion of young black SA CRC patients develop via the LS pathway due to earlier age onset and predominant MSH2/6 protein loss. SA patients of other ethnicities appear to follow the more well established sporadic MSI pathway.

## Introduction

Colorectal cancer (CRC) develops through three major molecular pathways namely: Chromosomal Instability (CIN), Microsatellite Instability (MSI), and the epigenetic instability or CpG island methylator phenotype (CIMP) pathway^1–3^. CIN or microsatellite stable (MSS) tumours account for 65–75% of CRC and mainly develop through mutant adenomatous polyposis coli (APC) gene with subsequent KRAS mutational activation, TP53 inactivation and somatic copy number alterations (SCNAs)^4,5^. MSI CRC occurs in approximately 15–16% of CRC due to inactivation of the DNA Mismatch Repair (MMR) system (MLH1, MSH2, MSH6, PMS2), with the majority (12%) presenting as sporadic MSI CRC as a result of epigenetic mutation (methylation silencing) of the promoter sequence of MLH1, and BRAF V600E oncogenic mutations^2,6–10^. The remaining subset (2–3%) is caused by germline mutations in either one of 4 MMR genes or the EpCAM gene and is a feature of Lynch syndrome (LS)^8,9,11–14^. CIMP CRC (10–20%) demonstrate hypermethylation of several promoter CpG island loci throughout the genome, leading to tumour suppressor and tumour-related gene inactivation. The CIMP and the MSI pathway overlaps in sporadic MSI CRCs, as these tumours display high levels of CIMP and MSI and are  characterized by a different type of precursor lesion in comparison to LS^15,16^. LS follows the classic adenoma-carcinoma sequence pathway as they present mainly with tubular adenomas (TAs) or tubulovillous adenomas (TVAs), whereas sporadic MSI CRCpremalignant lesions are sessile serrated adenomas (SSAs) developing through the serrated neoplasia pathway^15–17^. Clinicopathological features of sporadic MSI CRC include predominant occurrence in female patients, within the right colon and associated morphology includes signet ring cell and mucinous features with tumour infiltrating lymphocytes (TIL)^15,18–20^. LS patients are mainly associated with younger patients, with no gender preference and similar morphology as sporadic MSI tumours^12^.

Previous CRC studies conducted in SA showed a higher frequency of MSI CRC in young black patients through MMR deficiency than white patients^21–23^. These studies suggested a high frequency of LS, however additional validation studies and further molecular characterization of MSI CRC in black SA patients was recommended^24^.

MSI assessment has also shown important prognostic and predictive roles in CRC patient care, as MSI tumours are associated with a better prognosis than MSS tumours in early stage CRC, and treatment of MSI stage II tumours are not well responsive to 5-fluorouracil (5-FU) (standard treatment)^25,26^. MSI/BRAF wild-type tumours are also more suggestive of LS and is crucial for improving cancer surveillance and prevention screening for patient family members, due to their increased risk of developing cancer. Identifying LS patients and treating with aspirin (600 mg per day) have also shown to reduce the risk of CRC^27^.

This study assesses the frequency and features associated with MSI CRC over a 5-year period in a cohort of newly diagnosed CRC patients at the Charlotte Maxeke Johannesburg Academic Hospital (CMJAH) within SA. This data will provide insight into the CRC histopathological and molecular features associated with MSI CRC in black SA patients, with particular reference to MSI CRC frequency and the occurrence of suspected LS, a heretofore largely unassessed aspect of the disease.

## Methodology

### Patient demographics and tumour pathological characterisation

This was a retrospective study, comprising a 5-year cohort of 675 patients who had biopsy samples or colorectal resections fulfilling the histological criteria for adenocarcinoma of the colon or rectum from January 2011–December 2015, reported by the Charlotte Maxeke Johannesburg Academic Hospital (CMJAH) branch of the National Health Laboratory Service (NHLS)/Anatomical Pathology Division, Faculty of Health Sciences, University of the Witwatersrand. A total of 439 CRC cases with an MSI status by MMR immunohistochemistry (IHC) or MSI PCR result were included in this study. All cases were stratified by age, gender, ethnicity, tumour site, histological subtype, grade, stage (TNM classification by AJCC staging), presence and grade of precursor lesion, presence of tumour infiltrating lymphocytes (TIL) and Crohn’s-like inflammatory reaction (CIR). Tumour site was considered left-sided if distal from the splenic flexure and right-sided if proximal to the splenic flexure. This information was obtained from histology reports. A semi-quantitive H&E assessment using the Klintrup-Mäkinen score was used to determine the presence of TILs^28^. Immunophenotypic profiling of TILs was not performed. Family histories were not available from the histological reports. This research was conducted under the institution’s blanket ethics clearance (M10744) which allows for research to be carried out on all archived pathology specimens without informed consent from study participants. Additional study-specific ethics clearance was also obtained by the Ethics Committee of the University of the Witwatersrand (clearance number M120994), and all tests were performed according to the relevant guidelines and regulations.

### Molecular characterisation

**Figure 1:**
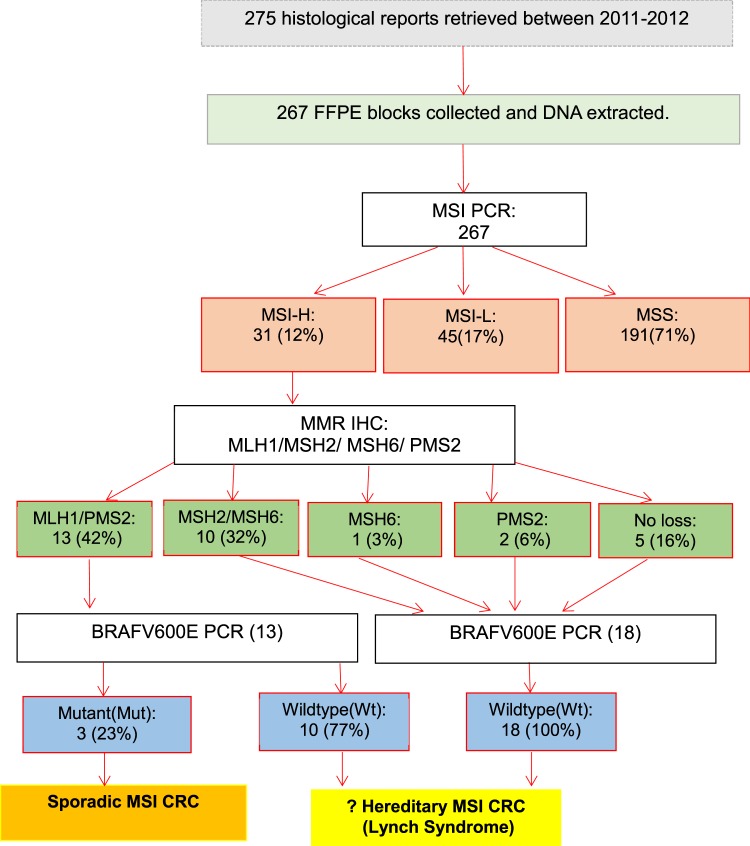
Flow diagram illustrating the molecular methodology and frequency results of CRC cases diagnosed between 2011–2012. MSI CRC was found in 12% of CRC cases, with predominant MLH1 loss, however only 23% of deficient MLH1 cases harboured BRAFV600E mutations. These findings suggest a large proportion of SA MSI CRC patients could develop through a hereditary pathway.

Molecular characterization of patient samples for frequency assessment was limited to the first 2 years (2011–2012) mainly due to the availability of funds (Figure 1). A total of 275 CRC cases were diagnosed during this period and a cohort of 267 archival CRC tumour specimens was retrieved, examined and subjected to molecular analysis. MSI PCR was conducted and samples with a MSI-High profile had additional BRAF V600E mutational analysis performed.

Cases without MMR IHC results as ascertained from the histology reports were screened for MMR protein loss by IHC.

DNA was extracted from 4 × 10µm sections of tumour tissue from formalin fixed paraffin embedded (FFPE) blocks using the QIAamp DNA FFPE tissue kit (Qiagen, Valencia, CA USA). The extracted DNA was subjected to a pentaplex PCR panel of 5 markers comprising mononucleotide repeats amplified by 5 sets of primers, i.e. (BAT-25, BAT-26, NR-21, NR-24, and NR-27) as described in Haghighi *et al*.^29^. Each antisense primer was labeled with 1 of the fluorescent markers FAM, HEX, or NED. The Applied Biosystems® True Allele® PCR master mix was used according to manufacturer’s instructions with slight modifications for successful amplification of the multiplex PCR. Fluorescent PCR products were subjected to capillary electrophoresis on the Applied Biosystems PRISM 3500 automated genetic analyzer, and allelic size was determined using Genemapper 4.0 software (Applied Biosystems). Samples with no allelic size variations in the 5 microsatellite markers were classified as microsatellite stable (MSS), allelic size variations found in 1 of the 5 microsatellites markers were classified as microsatellite instability-low (MSI-L) and variation in 2 or more microsatellite markers was classified as microsatellite instability-high (MSI-H)^29^. Previous studies have indicated black people show polymorphic variation frequencies within alleles NR21, BAT25 and BAT26 of approximately 10%^30–32^. For tumour samples that demonstrated instability in only 2 of these 3 markers, matched normal tissue was assessed to determine the instability status. In cases where no normal tissue could be assessed, samples were regarded as MSS.

MMR IHC was performed on MSI-H samples that were not previously assessed, utilizing the Dako EnVision™ FLEX detection system Kit and Autostainer Link 48 equipment (Dako, Glostrup, Denmark) according to manufacturer’s instructions. The primary monoclonal antibodies used were FLEX monoclonal mouse antibodies against MLH1 (clone ES05, ref ISO79, ready-to-use), MSH2 (clone FE11, ref IRO85, ready-to-use), MSH6 (clone EP49, ref IRO86, ready-to-use) and PMS2 (clone EP51, IRO87, ready-to-use) (Dako, Glostrup, Denmark). These were applied to 4-μm deparaffinized formalin fixed paraffin-embedded (FFPE) tissue sections. Blockage of endogenous peroxide activity was carried out with the EnVision™ FLEX Peroxidase-Blocking Reagent (Dako, Glostrup, Denmark) and Antigen retrieval performed at 97 °C for 20 minutes (pH 9.0). Antigen-antibody reaction was visualized with the DAB (Diaminobenzidine) solution, and subsequently counter-stained with hematoxylin. Each sample was screened by an expert pathologist for the absence or presence of expression of each of the 4 MMR proteins. Negative protein expression was defined as complete absence of nuclear staining within tumor cells. Expression of MMR proteins in normal epithelium, lymphocytes and stromal cells was used as positive internal controls for each case. The four deficient (d) MMR patterns assessed were 1: dMLH1/PMS2; 2: dMSH2/MSH6; 3: dMSH6 and 4: dPMS2.

All MSI-H samples were assessed for BRAF V600E mutations using the real-time Therascreen RGQ BRAF V600E mutation kit (Qiagen, Whitehead Scientific WHS), according to the manufacturer’s instructions. The kit uses ARMS^®^ and Scorpions^®^ technology, and includes a control assay to evaluate the adequacy of quality and quantity of DNA in a sample, before assessing the presence or absence of mutated DNA. Results were only deemed valid if the control run passed.

Data obtained from the molecular characterization study (2011–2012) led to a more comprehensive study which included cases with an MSI status from histological reports for the total 5-year study period (2011–2015).

### Statistical analysis

All clinical data was collected in an excel sheet and statistical analysis was performed using Stata Intercooled 7.0 (Stata, College Station, TX, USA) and Graphpad Prism version 7 (GraphPad Software, La Jolla, CA, USA). MSS and MSI-L cases were grouped together into one group (MSS), to establish unique associations with the counterpart MSI-H group. Patients were also analysed on the basis of ethnicity: black (African) versus other ethnic groups i.e., white (Caucasian), coloured (Mixed Ancestry), Indian (Asian); age and gender. The ability to draw statistically significant results on all ethnic groups was not possible due to small case numbers of certain groups. For this reason, White, Indian and Coloured patients were grouped into one “Other Ethnic Group” (OEG) to allow comparison with their black counterpart. Histopathological features were assessed as stated in the methodology section, and associations were evaluated using Fischer’s exact and Chi-square tests. A result with a P value less than 0.05 was considered statistically significant.

## Results

### Patient demographics

A total 439 of 672 CRC patients diagnosed between January 2011-December 2015 had MSI CRC status assessment. Of these cases, 247 (56%) were male and 192 (44%) female, with an overall median age of 58 years. Black patients contributed to 60% (262/439) of cases. (Column1 of Table 1).

**Table 1:** Descriptive analysis of CRC cases diagnosed at CMJAH between (2011–2015) with Microsatellite Instability status, stratified by CRC subtypes: MSI versus MSS.

	Number of cases (%)	CRC subtype		Statistical analysis: Significance indicated by*
Frequency:	All CRC cases	MSI	MSS	
2011–2015	439	61 (14)	378 (86)	
**Demographical data (2011–2015)**				
**GENDER**	**439**	**61**	**378**	P = 1.0000
Male	247 (56)	34 (56)	213 (56)	
Female	192 (44)	27 (44)	165 (44)	
**AGE**	**436**	**61**	**375**	**P** **=** 0.0033**
Min-Max	15–92	27–77	15–92	
Mean ± SD	56 ± 14	52 ± 13	57 ± 14	
Median	58	51**	59	
P25-P75 (Interquartile Range)	47–67	40–62	48–68	
95% CI	[55–58]	[48–55]	[56–59]	
**Categorical Age**	**436**	**61**	**375**	
≤50 years	137 (31)	29 (48)**	108 (29)	
>50 years	299 (69)	32 (52)	267 (71)	P = 0.0046**
**Ethnic groups**	**439**	**61**	**378**	P = 0.4858
Black	262 (60)	39 (64)	223 (59)	
Other Ethnic Groups (OEG)	177 (40)	22 (36)	155 (41)	
**Histological characteristics (2011–2015)**				
**TUMOUR SITE**	**430**	**60**	**370**	**P** **<** 0.0001***
Left (distal from splenic flexure)	273 (63)	12 (20)	261 (70)	
Right (proximal to splenic flexure)	154 (36)	47 (78)***	107 (29)	
Left and Right	3 (1)	1 (2)	2 (1)	
**TUMOUR SUBTYPE**	**430**	**60**	**370**	**P** **<** 0.0001***
Invasive Adenocarcinoma	384 (89)	45 (75)	339 (91)	
Mucinous Adenocarcinoma	30 (7)	13 (22)***	17 (5)	
Signet Ring Cell Adenocarcinoma	16 (4)	2 (3)	14 (4)	
**TUMOUR GRADE**	**371**	**45**	**326**	P = 0.1022
Low Grade	346 (93)	40 (89)	306 (94)	
Medium/High Grade	25 (7)	5 (11)	20 (6)	
**AJCC TNM STAGING**	**287**	**45**	**242**	P = 0.6226
I	11 (4)	2 (4)	9 (4)	
II	101 (35)	17 (38)	84 (35)	
III	158 (55)	22 (49)	136 (56)	
IV	17 (6)	4 (9)	13 (5)	
**TUMOUR INFILTRATING LYMPHOCYTES (TIL)**	**283**	**47**	**236**	**P** **=** 0.0045**
None	180 (64)	21 (45)	159 (67)	
Mild-moderate	103 (36)	26 (55)**	77 (33)	
**CHROHN’S LIKE INFLAMMATORY RESPONSE**	**283**	**47**	**236**	P = 1.0000
None	206 (73)	34 (72)	172 (73)	
Mild-moderate	77 (27)	13 (28)	64 (27)	
**LYMPHATIC INVASION**	**346**	**54**	**292**	P = 0.2582
Absent	242 (70)	34 (63)	208 (71)	
Present	104 (30)	20 (37)	84 (29)	
**POLYPS**	**313**	**49**	**264**	P = 0.6197
Absent	210 (67)	31 (67)	179 (68)	
Present	103 (33)	18 (33)	85 (32)	
**POLYP SUBTYPE**	**101**	**17**	**84**	**P** **=** 0.0216* TA vs TVA
Hyperplastic Polyp	3 (3)	0 (0)	3 (4)	
Pseudopolyp	2 (2)	1 (6)	1 (1)	
Sessile serrated Adenoma (SSA)	1 (1)	1 (6)	0 (0)	
Tubular Adenoma (TA)	53 (52)	4 (23)	49 (58)*	
Tubulovillous Adenoma (TVA)	42 (42)	11 (65)*	31 (37)	

### Pathological characteristics

Further descriptive analysis was done with respect to histological characteristics, with left sided tumours accounting for the majority of cases (63%; 273/430) (Column1 of Table 1). The most common subtype was invasive adenocarcinoma (384/430; 89%), followed by invasive mucinous adenocarcinoma (30/430; 7%). AJCC tumour stage II and III accounted for the majority of cases (259/287; 90%) (Table 1). Further stratification by MSI and MSS subtype is illustrated in the 2^nd^ and 3^rd^ column in Table 1.

### MSI CRC molecular characterization (2011–2012)

MSI PCR performed on 267 cases (2011–2012) illustrated the majority were MSS (191/267; 71%), followed by MSI-L (45/267; 17%) and MSI-H (31/267; 12%). MMR IHC results demonstrated dMLH1 in most MSI CRC cases (13/31; 42%), followed by dMSH2/6 (10/31; 32%), dPMS2 (2/31; 6%) and dMSH6 (1/31; 3%). BRAF mutational analysis was performed on all MSI-H cases with only 10% (3/31) harbouring a mutation, and all 3 (3/13; 23%) occurred in dMLH1 tumours (See Fig. 1**)**.

### MSI versus MSS CRC subtyping (2011–2015)

Male patients comprised 56% of the entire CRC cohort. Correlation between subtype and age was found (P = 0.0033) as median age was significantly lower in MSI CRC compared to the MSS subtype (51 vs 59 years respectively) (Table 1**)**. A higher frequency of MSI tumours was seen in blacks ([22/148; 15%] versus OEG patients [9/119; 8%]), for the period 2011–2012. A significant relationship was seen between CRC subtype and tumour site (P < 0.0001), as MSS was associated with left sided tumours (261/370; 70%) and MSI with the right sided tumours [(47/60; 78%). A link between CRC molecular subtype and tumour subtype was also found (P < 0.0001), as mucinous adenocarcinoma occurred at a more frequent rate in MSI CRC; (13/60; 22%) than MSS CRC (17/370; 5%). Tumour infiltrating lymphocytes (TIL) response and subtype was found to have a correlation (P = 0.0045) as this occurred more often in the MSI group (26/47; 55%) than the MSS (77/236; 33%). Polyp subtypes tubular adenoma (TA) and tubulovillous adenoma (TVA) were associated with molecular subtypes (P = 0.0216) as TAs occurred more frequently in MSS CRC (49/84; 58%) and TVAs in MSI CRC (11/17; 65%).

Within the MSI CRC subgroup, additional association evaluations between ethnicity, gender, tumour site, defective MMR proteins and BRAFV600E mutation status were performed **(**Table 2**)**. A correlation with age was shown (P = 0.0006) as black patients were significantly younger compared to OEG patients (median age: 47 versus 62, respectively). Tumour site and ethnicity showed an association for 2011–2012 data (P = 0.0105), as the majority of tumours were right sided within OEG patients (8/9; 89%), whereas black patients had left (8/22; 36%) and right-sided tumours (13/22; 59%) (Supplementary Data Table 2). Data for 2011–2015 however showed no such association (P = 0.1817). An association between ethnic groups and dMMR protein expression was found (P = 0.0098), where dMLH1/PMS2 expression was more commonly seen in OEG patients (15/22; 68%), and dMSH2/6 in black patients (19/39; 49%). BRAF V600E mutation status and ethnicity were related in the 2011–2012 data (P = 0.0187) (Supplementary Data Table 2), as all black patients were wild-type for BRAF gene status, however within the OEG, BRAF mutations were only found in dMLH1 tumours (3/5; 60%). For the 2011–2015 BRAFV600E data analysis, 2011–2012 results were dominantly represented with an additional 3 cases from 2013–2015 reports with BRAFV600E status. An increased association between BRAFV600E and OEG patients was shown (P = 0.0045) for this period.

**Table 2:** Descriptive analysis of MSI CRC diagnosed at CMJAH (2011–2015), stratified by ethnic groups: Black versus Other Ethnic Groups (OEGs).

		MSI CRC stratified by ethnic groups (2011–2015)		
		No. of cases (%)		
Demographic Data	Number of cases (%)	Black	OEGs	Statistical Significance
**GENDER**	**61**	**39**	**22**	P = 0.5946
Male	34 (56)	23 (59)	11 (50)	
Female	27 (44)	16 (41)	11 (50)	
**AGE**	**61**	**39**	**22**	P = 0.0006***
Median	51	47***	62	
Min-Max	27–77	28–74	27–77	
Mean ± SD	52 ± 13	47 ± 11	59 ± 14	
P25-P75	40–62	38–55	48–70	
95% CI	[48–55]	[44–51]	[53–65]	
**Categorical Age**	**61**	**39**	**22**	P = 0.0316*
≤50 years	29 (48)	23 (59)*	6 (27)	
>50 years	32 (52)	16 (41)	16 (73)*	
**TUMOUR SITE**	**60**	**39**	**21**	P = 0.1817 Left vs Right
Left	12 (20)	10 (26)	2 (10)	
Right	47 (78)	28 (72)	19 (90)	
Left and Right	1 (2)	1 (2)	0 (0)	
**MMR PROTEIN IHC**	**61**	**39**	**22**	P = 0.0098** MLH1/PMS2 vs MSH2/MSH6
dMLH1/PMS2	31 (51)	16 (41)	15 (68)**	
dMSH2/MSH6	22 (36)	19 (49)**	3 (14)	
dMSH6	1 (2)	1 (2.5)	0 (0)	
dPMS2	2 (3)	1 (2.5)	1 (4)	
pMMR	5 (8)	2 (5)	3 (14)	
*BRAF V600E	**34**	**24**	**10**	P = 0.0045**
Wildtype	30 (88)	24 (100)**	6 (60)	
Mutation	4 (12)	0 (0)	4 (40)**	

Left (Colon distal  from splenic flexure); Right (Colon proximal to splenic flexure); Microsatellite Instability (MSI); Mismatch repair (MMR); Deficient (d); Proficient (p). Significance indicated by an asterix.

*Majority of the samples screened for BRAFV600E mutations (31/34; 91%) were from the 2011–2012 cohort. The remaining 3 were retrieved from histological reports for the remaining period (2013–2015).

Table 3 illustrates a multivariate analysis including variables considered clinically significant for MSI status. Total number of observations were 277. Age, tumour site, mucinous adenocarcinomas and TILS associated after controlling for confounding factors. Patients >50 years were 67% less likely to have MSI and right sided tumours were 8X more likely to have MSI. Invasive adenocarcinomas were 73% less likely to show MSI than mucinous adenocarcinomas. Absence of a TIL response was 60% most likely not to have MSI in comparison to those with TILs. TVAs were not included due to the small sample size, reducing the power and value of the analysis.

**Table 3:** Multiple logistic regression factors at risk for MSI CRC.

Variable	Multivariate	
	Odds ratio with CI	P-Value
**Gender**		
Male	0.834 (0.394, 1.762)	0.634
**Age Category**		
>50 years	0.330 (0.146, 0.745)	0.008**
**Ethnic Group**		
Black	0.902 (0.411, 1.983)	0.799
**Tumour Site**		
Right	7.661 (3.150, 18.630)	0.000***
**Subtype**		
Invasive Signet ring cell	0.971 (0.007, 1.249)	0.074
Invasive Adenocarcinoma	0.270 (0.099, 0.731)	0.010*
**Grade**		
LG	0.535 (0.142, 2.007)	0.354
**Tumour Infiltrating Lymphocytes**		
None	0.392 (0.182, 0.845)	0.017*
**Lymphatic Invasion**		
Present	0.829 (0.376, 1.825)	0.642

Number of observations were 277. Young Patients under 50 years, with right-sided tumours, displaying mucinous features with TILs were more likely to have MSI. Significance indicated by an asterix.

## Discussion

MSI CRC was detected in 12% of CRC patients diagnosed during the period 2011–2012, however when stratified by ethnic groups, a higher frequency was found in black versus OEG patients although only of borderline significance (15% versus 8% respectively; P = 0.0831). Black patients with MSI tumours occurred in younger patients with left- (36%) and right-sided (59%) tumours, associated with dMSH2/6 (45%), and dMLH1/PMS2 (36%) tumours with a lack of BRAF mutations, indicating a possible LS cause^10,33–35^. MSI CRC in Western population groups are shown to dominantly occur in female patients with right-sided tumours, a serrated morphology and BRAFV600E mutations, MLH1 protein loss due to promoter methylation and a high CIMP molecular phenotype^2,20^.

In the SA cohort, MSI CRC was associated with younger age, right sided tumours, mucinous adenocarcinomas, TIL response, and TVA precursor lesions. This correlates well with findings in the literature^36,37^. Histopathological features were further stratified by ethnic groups (black versus OEG) to assess if any associations occurred with regard to gender, age, tumour site, MMR protein expression profile and BRAF mutation status. Within the 2011–2012 cohort, patients within the OEG were predominantly older (median age: 62), female (67%), possessed right sided tumours (89%), with deficient MLH1 (56%) expression, of which 60% harboured BRAF mutations. The 2011–2015 cohort however demonstrated an equal ratio of male to female patients, which could signify an under-diagnosis of female patients with MSI within the OEG. All other variables illustrated increased statistical associations within the 5-year cohort study. Collectively these features suggest the majority of OEG patients follow the more well established sporadic MSI CRC pathway^19,20^. Within the black patient group, all 2011–2012 data analysed with statistical significant associations revealed increased significance within the 5 year cohort, with the exception of tumour site where 2011–2012 data illustrated significance with an increased incidence of left-sided MSI tumours. This could possibly be due to pathologists generally screening more right than left-sided tumours for MSI CRC.

MSI CRC also showed a high percentage of proficient (p) MMR expression (16%) for the universal 2-year screen, despite high concordance rates (95–96%) for MSI PCR versus IHC reported^38^. This could possibly be indicative of aberrant non-functional proteins expressed or an alternative MMR protein involved in the development of MSI in this group of patients. This would suggest the 5 mononucleotide panel for MSI testing is the more sensitive tool for detecting instability in the South African setting. Perhaps this could be explored further for alternative causes of dMMR in these patients. For example, MSH3 loss of expression has recently been shown to be associated with MSI in African American patients^39,40^.

Multiple logistic regression analysis was also conducted to control for the effect of confounding factors, young age (≤50 years), right-sided tumours, mucinous adenocarcinomas and TILS were associated with MSI CRC in SA patients.

Limitations of the study included patient selection bias as patients from the 2013–2015 cohort were only selected if an MSI status was available. In addition, only a few patient samples with available data in certain subgroup analysis, such as TNM stage of disease, TILs, polyp subtype could be analysed, and this is mainly due to biopsy samples also included in the cohort. Only a subset of MSI samples (34/61; 56%) for the period 2011–2015 were included for BRAFV600E analysis, these largely from cases between period 2011–2012. This also represents a limitation of the study. The majority of patients diagnosed at CMJAH are of the black population group, therefore selection bias could play a role in the selection effect due to smaller sample numbers in the comparator OEG patient group. Previous MSI studies in SA however, have largely shown MLH1 mutations as the cause of hereditary and sporadic CRC in OEG patients (specifically the coloured and white population groups in the Western and Northern Cape regions of SA)^23,41,42^. A study carried out by Cronje *et al*. in 2009 at CMJAH in Johannesburg demonstrated a large number of young black patients had increased loss of MMR protein expression, particularly MSH2, in comparison to their white counterpart, also suggesting a heritable cause^21^. Findings from this study confirm dMLH1 and dMSH2 associations within young OEG and black SA patients respectively.

**Figure 2:**
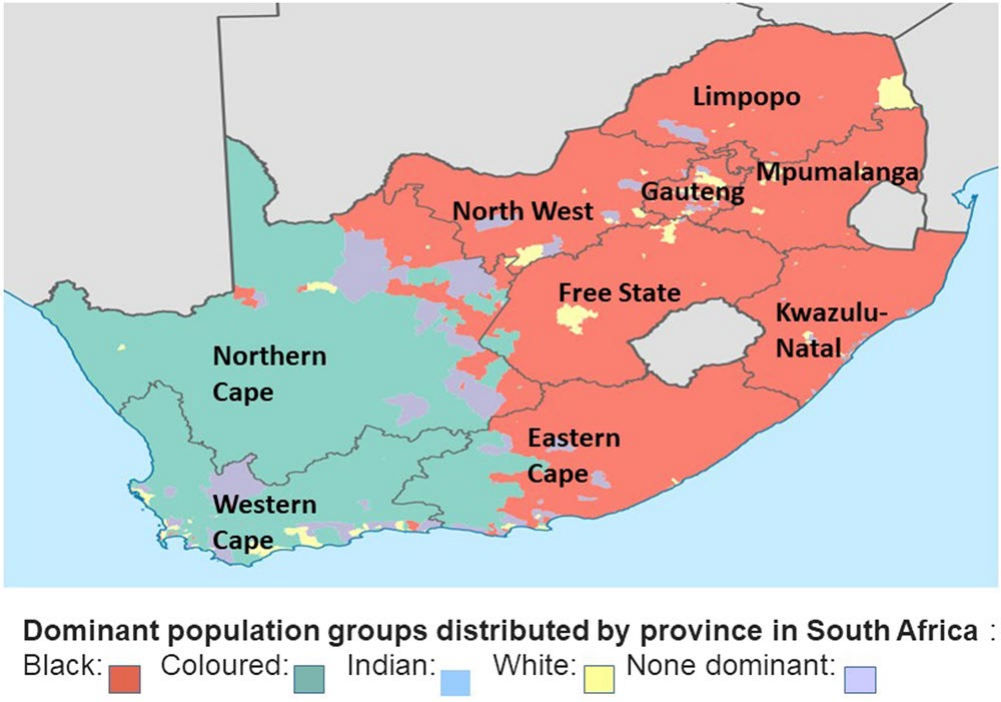
Map of South Africa illustrating the distribution of population groups by the different provinces, (adapted from the Wikipedia map of South Africa 2011)^54^.

South Africa is comprised of 9 provinces, with an estimated mid-year population of 58.78 million. The Northern Cape comprises 2.2% of the population, Western Cape 11.5%, and Gauteng 25.8%^43,44^. The Western and Northern Cape comprises mainly of the coloured population within SA, and LS have largely been identified in these 2 provinces (See Fig. 2)^23,41,45^. Genetic services are offered to newly diagnosed LS patients and family members in these regions mainly through academic centres based in the Western Cape, and to smaller surrounding towns and rural areas through outreach programmes^23,41,45,46^.

The Gauteng province, although the smallest, is the most densely populated and urbanized with nearly 700 people per square kilometre^43,44^. This is largely due to the discovery of gold in Johannesburg during the 1800’s, with a large influx of migrants from Africa and Europe^46^. The black population is the predominant group (77.4%) followed by white (15.6%) in this region^43,44^. LS genetic screening in Gauteng and remaining 6 provinces have been poor to non-existent, even though preliminary findings have suggested a possible hereditary cause, particularly in black patients from the 1990’s^21,47–49^.

The healthcare financial system in SA is based on private and public funding, with the majority (57%) of the population utilizing public healthcare services, however 80% of healthcare expenditure in SA occurs in the private sector^46,50^. Genetic services such as testing and counselling are mostly offered in academic centres, and subsidies for these services are limited primarily due to the high medical costs associated with the HIV/AIDS epidemic^46^. Pathologists within the National Health Laboratory Services include a possible hereditary cause in histopathological reports of these patients with suspected LS, but genetic services are limited to counselling, particularly within the public sector of the Gauteng province. Colonoscopic surveillance of family members at risk for LS is difficult and expensive in a resource-poor country, however previous LS studies carried out within the Western Cape region of SA have shown the identification of the causative-mutation simplifies and reduces costs of surveillance considerably^22,45,51^.

If LS is the more prevalent MSI subtype in black SA patients, identifying the causative mutation together with regular colonoscopy screening is recommended for all affected individuals and families by the 2^nd^ decade of life, to be repeated every 1–2 years^52^. LS patients treated with aspirin have a considerably reduced risk and reduced mortality rates associated with CRC^27,53^.

## Conclusion

This study revealed MSI in black SA CRC patients is associated with earlier age onset, higher frequency of MSH2/6 loss and a lack of BRAFV600E mutations, suggesting Lynch or “Lynch-Like Syndrome” (LLS) as the main hereditary driver. Implementation of universal MSI screening is recommended as a routine diagnostic test within the SA setting to increase the detection rate of LS or LLS. This data warrants further molecular investigations in defining the underlying germline or somatic mutations. Appropriate early detection, surveillance and prevention strategies will reduce the risk and improve overall management of the disease in suspected LS patients and affected family members, particularly in the black population group in SA.

### Compliance with ethical standards

This was a retrospective study on already available biological material and ethics clearance was obtained from the Human Ethics Research Committee, Faculty of Health Sciences: Ethics clearance reference number: M120994.

## Supplementary information

Supplementary information

## Data Availability

The dataset generated and analyzed during the current study are available from the corresponding author on reasonable request.

## Supplementary information

is available this paper at 10.1038/s41598-019-51316-4.
