# Supplementary material for: Microsatellite Instability assessment in Black South African Colorectal Cancer patients reveal an increased incidence of suspected Lynch syndrome
Source: Sci Rep. 2019 Oct 21;9:15019. doi: 10.1038/s41598-019-51316-4 (PMC6803663; doi:10.1038/s41598-019-51316-4)
Supplement: Supplementary file 1 — Supplementary information [file 41598_2019_51316_MOESM1_ESM.pdf]

Microsatellite Instability assessment in Black South African Colorectal Cancer patients reveal an increased incidence of suspected Lynch syndrome.

Authors:

McCabe M.[1], Perner Y.[1], Magobo R.[1], Magangane P.[1], Mirza S.[2], Penny C.[2]

[1]Department of Anatomical Pathology, School of Pathology, Faculty of Health Sciences, University of the Witwatersrand, Parktown, Johannesburg, 2193

[2]Department of Internal Medicine, Faculty of Health Sciences, University of the Witwatersrand, Parktown, Johannesburg, 2193.

Table 1: Descriptive analysis of CRC cases diagnosed at CMJAH between (2011-2012), stratified by CRC subtype: MSI versus CIN:

	**Number of cases (%)**	**CRC subtype**		**Statistical analysis:**
	**All CRC cases**	**MSI**	**MSS**	
**Frequency/ Prevalence**	**267**(100)	31(12)	236(88)	
**Demographical data**				
**GENDER**	**267**	**31**	**236**	P= 0.5662
Male	145 (54)	15(48)	130(55)	
Female	122 (46)	16(52)	106(45)	
**AGE**	**266**	**31**	**236**	**P= 0.0209 ***
Min-Max	20-92	32-77	20-92	
Mean ± SD	58±14	53 ±12	59 ±14	
Median	59	51	60	
P25-P75 (Interquartile Range)	48-69	46-62	50-69	
95% CI	[56-60]	[48-58]	[57-60]	
**RACE**	**267**	**31**	**236**	P = 0.0831
Black	148(55)	22(71)	126(53)	
Other Race Group	119(45)	9(29)	110(47)	
**Histological characteristics**				
**TUMOUR SITE**	**265**	**31**	**234**	**P < 0.0001 *****
Left	191(72)	9(29)	182(78)	
Right	72(27)	21(68)	51(22)	
Left and Right	2(1)	1(3)	1(0)	
**TUMOUR SUBTYPE**	**261**	**31**	**230**	**P = 0.0044 ****
Invasive Adenocarcinoma	232(89)	22(71)	210(91)	
Mucinous Adenocarcinoma	23(9)	8(26)	15(7)	
Signet Ring Cell Adenocarcinoma	6(2)	1(3)	5(2)	
**TUMOUR GRADE**	**219**	**22**	**197**	**P = 0.0498***
Low Grade	204(93)	18(82)	186(94)	
Medium/ High Grade	15(7)	4(18)*	11(6)	
**AJCC TNM STAGING**	**150**	**24**	**120**	P= 0.8407
I	7(6)	2(7)	7(6)	
II	54(36)	8(30)	46(37)	
III	79(53)	12(56)	67(52)	
IV	8(5)	2(7)	6(5)	
**TUMOUR INFILTRATING LYMPHOCYTES (TIL)**	**149**	**24**	**125**	**P = 0.0045****
None	96(64)	9(37)	87(70)	
Mild-moderate	53(36)	15(63)	38(30)	
**CHROHN'S LIKE INFLAMATORY RESPONSE**	**149**	**24**	**125**	P = 0.7978
None	112(64)	19(79)	93(74)	
Mild-moderate	37(36)	5(21)	32(26)	
**LYMPHATIC INVASION**	**207**	**30**	**177**	**P = 0.0413 ***
Absent	156(75)	18(59)	138(79)	
Present	51(25)	12(41)	39(21)	
**POLYPS**	**166**	**23**	**143**	P = 0.8149
Absent	107(64)	14(61)	93(65)	
Present	59(36)	9(39)	50(35)	
**POLYP SUBTYPE**	**56**	**9**	**47**	P = 0.4466
Hyperplastic Polyp (HP)	2(3)	0(0)	2(4)	
Pseudopolyp (PSP)	1(2)	0(0)	1(2)	
Sessile serrated Adenoma (SSA)	1(2)	1(11)	0(0)	
Tubular Adenoma (TA)	28(50)	3(33)	25(53)	
Tubulovillous Adenoma (TVA)	24(43)	5(56)	19(41)	

Table 1: Microsatellite Instability (MSI); Microsatellite stable (MSS). Significance indicated by an asterix.

Table 2: Descriptive data analysis of MSI CRC diagnosed at CMJAH (2011-2012), stratified by race groups: Black versus Other.

		**MSI CRC Stratified by race groups (2011-2012) No. of cases (%)**		
**Demographic Data**	**Number of cases (%)**	**Black (B)**	**Other (O)**	**Statistical Significance**
**GENDER**	**31**	**22**	**9**	P=0.4331
Male	15(48)	12(55)	3(33)	
Female	16(52)	10(45)	6(67)	
**AGE**	**31**	**22**	**9**	P=0.0451*
Median	51	50*	62	
Min-Max	32-77	32-74	46-77	
Mean ± SD	53 ±12	50±11	61±12	
P25-P75	46-62	40-56	48-73	
95% CI	[48-58]	[45-55]	[51-70]	
**Categorical Age**	**31**	**22**	**9**	P=0.7384
≤50years	14 (45)	11(50) *	3(33)	
>50 years	17 (55)	11(50)	6(67) *	
**TUMOUR SITE**	**31**	**22**	**9**	**P = 0.0105 ***<br>Left vs Right
Left	9(29)	8(36)	1(11)	
Right	21(68)	13(59)	8(89)	
Left and Right	1(3)	1(5)	0(0)	
**MMR PROTEIN IHC**	**31**	**22**	**9**	**P = 0.0411***<br>MLH1 vs MSH2/6
dMLH1/PMS2	13(42)	8(36)	5(56) *	
dMSH2/MSH6	10(32)	10(45) *	0(0)	
dMSH6	1(3)	1(5)	0(0)	
dPMS2	2(6)	1(5)	1(11)	
MMR proficient	5(16)	2(9)	3(33)	
**BRAF V600E**	**31**	**22**	**9**	**P = 0.0187***
Wildtype	28(90)	22(100)	6(67)	
Mutation	3(10)	0(0)	3(33)*	

Table 2: Microsatellite Instability (MSI); Mismatch repair (MMR). Deficient (d). Proficient (p). Significance indicated by an asterix.
